# Physician Self-assessment of Shared Decision-making in Simulated Intensive Care Unit Family Meetings

**DOI:** 10.1001/jamanetworkopen.2020.5188

**Published:** 2020-05-19

**Authors:** Scott T. Vasher, Ian M. Oppenheim, Pragyashree Sharma Basyal, Emma M. Lee, Margaret M. Hayes, Alison E. Turnbull

**Affiliations:** 1 Department of Internal Medicine, Division of Hospital Medicine at Johns Hopkins Bayview Medical Center, Johns Hopkins University School of Medicine, Baltimore, Maryland; 2 Division of Pulmonary and Critical Care Medicine, Johns Hopkins University School of Medicine, Baltimore, Maryland; 3 Division of Geriatric Medicine and Gerontology, Johns Hopkins University School of Medicine, Baltimore, Maryland; 4 Outcomes After Critical Illness and Surgery (OACIS) Group, Johns Hopkins University School of Medicine, Baltimore, Maryland; 5 Division of Pulmonary, Critical Care, and Sleep Medicine, Beth Israel Deaconess Medical Center, Harvard Medical School, Boston, Massachusetts; 6 Shapiro Institute for Education and Research at Beth Israel Deaconess Medical Center, Harvard Medical School, Boston, Massachusetts; 7 Department of Epidemiology, Johns Hopkins Bloomberg School of Public Health, Baltimore, Maryland

## Abstract

**Importance:**

Professional guidelines have identified key communication skills for shared decision-making for critically ill patients, but it is unclear how intensivists interpret and implement them.

**Objective:**

To compare the self-evaluations of intensivists reviewing transcripts of their own simulated intensive care unit family meetings with the evaluations of trained expert colleagues.

**Design, Setting, and Participants:**

A posttrial web-based survey of intensivists was conducted between January and March 2019. Intensivists reviewed transcripts of simulated intensive care unit family meetings in which they participated in a previous trial from October 2016 to November 2017. In the follow-up survey, participants identified if and how they performed key elements of shared decision-making for an intensive care unit patient at high risk of death. Transcript texts that intensivists self-identified as examples of key communication skills recommended by their professional society’s policy on shared decision-making were categorized.

**Main Outcomes and Measures:**

Comparison of the evaluations of 2 blinded nonparticipant intensivist colleagues with the self-reported responses of the intensivists.

**Results:**

Of 116 eligible intensivists, 76 (66%) completed the follow-up survey (mean [SD] respondent age was 43.1 [8.1] years; 72% were male). Sixty-one of 76 intensivists reported conveying prognosis; however, blinded colleagues who reviewed the deidentified transcripts were less likely to report that prognosis had been conveyed than intensivists reviewing their own transcripts (42 of 61; odds ratio, 0.10; 95% CI, 0.01-0.44; *P* < .001). When reviewing their own transcript, intensivists reported presenting many choices, with the most common choice being code status. They also provided a variety of recommendations, with the most common being to continue the current treatment plan. Thirty-three participants (43%) reported that they offered care focused on comfort, but blinded colleagues rated only 1 (4%) as explaining this option in a clear manner.

**Conclusions and Relevance:**

In this study, guidelines for shared decision-making and end of life care were interpreted by intensivists in disparate ways. In the absence of training or personalized feedback, self-assessment of communication skills may not be interpreted consistently.

## Key Points

**Question:**

Does intensivist self-assessment of communication skills endorsed by professional guidelines align with assessments of the same skills by blinded expert colleagues?

**Findings:**

In this survey, 76 US intensivists read deidentified transcripts from their intensive care unit family meeting and rated themselves as conveying prognosis, highlighting choice, providing recommendations and offering the option of care focused on comfort. Sixty-one of 76 intensivists reported conveying prognosis, and blinded colleagues agreed that 42 of those 61 had conveyed the patient’s risk of death.

**Meaning:**

Clinicians who lack communication skills endorsed in professional guidelines may also lack the metacognitive skills required to recognize their deficiencies, which makes routine feedback and continuing education on communication and shared decision-making essential at all levels of practice.

## Teaser

This survey study assesses how the self-assessments of intensivists who read a transcript of their participation in an intensive care family meeting simulation compare with assessments of blinded colleagues reading the same transcript in reporting the performance of key communication skills endorsed by their professional societies.

## Introduction

For 2 decades, treatment in intensive care units (ICUs) during the last month of life has increased among Americans age 65 years and older,^1^ including among people with advanced dementia who are unlikely to live longer as a consequence of being in an ICU.^2^ Although intensive care at the end of life is common, many people would prefer to prioritize comfort. To help ensure that critically ill patients and their families can choose care that aligns with their values and goals, critical care societies encourage ICU physicians (intensivists) to practice shared decision-making regarding preference-sensitive interventions.^3^

Determining how doctors are interpreting and implementing recommendations about shared decision-making in the ICU presents a challenge. When family meetings are recorded, analyses are usually performed by clinical experts who review written transcripts, audio recordings, or video of family meetings for research purposes.^4,5,6,7,8,9,10,11,12^ This method of studying communication is efficient and practical but may not reflect how intensivists experience or interpret a conversation. For a recommendation or guideline to change practice, clinicians must either be able to independently determine whether they are following it or receive feedback on adherence.^13^

To understand how intensivists self-evaluate their shared decision-making skills, we asked 116 attending intensivists from US hospitals to review the transcript of a simulated ICU family meeting they participated in as part of a randomized clinical trial.^14^ Intensivists who agreed identified both if and how they performed key elements of shared decision-making for an ICU patient at high risk of death. We then characterized the text that intensivists self-identified as examples of key communication skills recommended by their professional society’s policy on shared decision-making.^3^ We also compared intensivist self-evaluation with the evaluations of blinded colleagues reviewing the same transcripts for research purposes.

## Methods

### Study Design and Participants

We conducted a survey study that followed up intensivists who had participated in the Simulated Communication with ICU Proxies (SCIP) trial (NCT02721810).^14^ Briefly, SCIP was a randomized clinical trial that enrolled 116 attending intensivists from 17 states to participate in a medical simulation between October 2016 and November 2017. Participants provided written informed consent and received $500 in compensation. Enrolled intensivists individually reviewed the medical record of a hypothetical 81-year-old patient on ICU day 3, with an estimated risk of mortality of 88% based on the Mortality Probability Model II-72 hours^15,16^ despite appropriate medical management. Each enrolled intensivist then participated in an audio- and video-recorded simulated family meeting with an actor portraying the hypothetical patient’s daughter and surrogate decision maker. Intensivists were instructed to interact with the daughter however they felt was appropriate given the clinical scenario. The actor was instructed to portray a daughter who was passive, had low health literacy, and would accept physician recommendations. Encounter recordings were transcribed verbatim and deidentified. This study was approved by the Johns Hopkins Medicine Institutional Review Board. Completion of the survey served as informed consent. This study followed the American Association for Public Opinion Research (AAPOR) reporting guideline for survey studies.

### Survey Design and Distribution

In January 2019, at least 1 year after they had participated in the original trial, all SCIP trial participants were recruited via email to complete a follow-up survey. The survey asked the trial participants to identify key elements of shared decision-making in their own transcribed family meeting from the SCIP trial. The elements were drawn from a joint policy statement issued by The American College of Critical Care Medicine and American Thoracic Society in 2016 on shared decision-making in ICUs.^3^ The policy statement endorsed key communication skills including disclosing prognosis, highlighting choice, and providing a recommendation. Participants’ self-evaluated performance of these guidelines was assessed via the following 3 survey questions: “During the simulation, did you (1) convey prognosis for risk of death? (2) highlight that there is a choice? and (3) provide a recommendation?”

Additionally, the Critical Care Choosing Wisely Task Force recommends offering the option of care focused entirely on comfort for patients at high risk for death or severely impaired functional recovery.^17^ To assess how intensivists implemented this recommendation, study participants were asked a fourth question: During the simulation, did you offer the alternative of care focused entirely on comfort? All 4 survey questions had the following response options: done, not done, or not applicable. If an intensivist marked a communication skill as done, they were instructed to mark all text in their transcript corresponding to the performed skill. Intensivists received $50 for returning their questionnaire and annotated transcript.

We excerpted the text marked in simulation transcripts in response to 2 of the survey questions: (1) During the simulation did you highlight that there is a choice? and (2) During the simulation did you provide a recommendation? Two of us (I.M.O. and E.M.L.) reviewed these excerpts and inductively created categories to describe common response patterns. Category definitions were discussed with all authors and further refined. Differences were resolved through iterative discussion until all authors agreed on definitions for each category. Two of us (S.T.V. and M.M.H.) then reread and categorized all excerpts with some excerpts fitting multiple categories.

As part of the original SCIP trial analysis the deidentified transcripts from each simulated encounter were reviewed by 2 blinded nonparticipant intensivist colleagues. These colleagues evaluated with a yes or no response whether intensivists conveyed prognosis for risk of death during the simulated family meeting. The colleagues also evaluated the clarity with which intensivists offered the option of care focused on comfort with responses of not offered, not understandable, vague, understandable, or clear. Interreviewer differences were reconciled via discussion.^14^ We compared these evaluations with intensivists’ self-reported responses to the same questions collected via survey more than a year later.

### Statistical Analysis

We used descriptive statistics to characterize enrolled participants and summarized deductively coded response categories using counts and proportions. To test whether self-assessment differed from assessments by blinded colleagues when deciding whether prognosis had been conveyed, we estimated an odds ratio using an exact version of the McNemar test^18,19^ for paired binary response data because of small count size in some cells. A 2-sided *P*<.05 was considered statistically significant. Data were analyzed using R, version 3.6.0 (R Project for Statistical Computing).

## Results

The SCIP trial included 116 participants from 17 states.^14^ Follow-up surveys were completed and returned by 76 (66%) intensivists. The mean (SD) respondent age was 43.1 (8.1), 72% were male, 76% identified as white (non-Hispanic), and first residency was completed a mean (SD) of 13.7 (8.5) years prior to the simulation. There were no statistically significant differences in age, sex, race/ethnicity, or years since training when comparing intensivists who participated in vs those who declined to participate in the follow-up study (Table 1).

**Table 1.** Intensivist Demographic Characteristics Stratified by Follow-up Study Participation

Characteristic	Participated in follow-up, No. (%)		*P* value^a^
	No	Yes	
Participants, No.	40	76	
Age, mean (SD)	40.5 (8.4)	43.1 (8.1)	.10
Male	25 (63)	55 (72)	.38
Race/ethnicity^b^			
Asian	12 (30)	11 (15)	.24
Black or African American	1 (3)	3 (4)	
More than 1 race/ethnicity	3 (8)	3 (4)	
White	23 (58)	58 (76)	
For-profit hospital	8 (20)	5 (7)	.06
University hospital	27 (68)	54 (71)	.85
Time since completing first residency, mean (SD), y	11.7 (8.6)	13.7 (8.5)	.23
Time worked in the ICU in the past year, mean (SD), wk	18.6 (12.4)	15.4 (10.8)	.16

Abbreviation: ICU, intensive care unit.

^a^ Hypothesis testing of categorical variables was performed with χ^2^ tests and continuous variables with *t* test.

^b^ Results do not sum to total number because 1 intensivist in each group preferred not to respond.

### Qualitative Analysis of Highlighting Choice

Thirty-two intensivists (42%) responded yes to the question, “During the simulation, did you highlight that there is a choice?” and no to the question, “During the simulation, did you offer care focused on comfort?” Representative text marked as highlighting choice by these 32 intensivists is presented in Table 2. The most common choice intensivists offered involved code status (34%). Intensivists commonly presented this choice by contrasting cardiopulmonary resuscitation (CPR) and natural death: “There's um… [a] couple of things we could do if his heart would stop beating. One is we could do CPR. And try to bring him back. The other option is to allow him to… allow him to go naturally if his heart would stop on its own.”

**Table 2.** Categorized Choices Identified in Text Marked by 32 Intensivists Reviewing Their Own Transcripts Who Responded Yes to the Question, “During the Simulation, Did You Highlight That There Is a Choice?”

Response category^a^	No. (%)^b^	Intensivist response example
Option to withhold CPR (DNR)	11 (34)	“And so what I'd like for you to think about over the next day or two is if your dad continues to get worse over the next couple of days, and if his heart were to stop beating, should we attempt to restart it?”
Continuing or adding to current life support	5 (16)	“…would he want dialysis, do you think?”
Choice is unclear or ambiguous	3 (9)	“And I just want you to know that we're going to continue to treat these things aggressively, but there might be point where you and the rest of your family think that we're doing too much, and if you ever think we're doing too much to him that's not going to benefit him, please let us know.”
Multiple options rather than 1 choice	2 (6)	“… if he wasn't able to get off the ventilator… would he find it acceptable to have tracheotomy tube… a tube here instead of attaching the machine to the tube in his mouth… and whenever we do that, we have to be able to feed you... we would have to put a feeding tube in through the stomach in order to feed you... You know, you can't manage the machine at home… So that does mean going to a facility…”
Care focused on comfort in the future	2 (6)	“…you and I will sit down and have a discussion as to whether our care goals change, okay? And whether instead of trying to prolong his life and survival, whether we shift to making sure that he is absolutely comfortable.”
Consultation to palliative care team	1 (3)	“We have people in the hospital whose specialty is making people comfortable and that doesn't mean that they can't do that also while a patient is still being treated with aggressive medical care with a goal of survival, they can work with us. They're called a palliative care team.”
No choice is offered yet	14 (44)	“If he's not getting better or certainly if he's getting worse over the next few days or a week or so, we're going to certainly have to sit down again and decide how to deal with things.”

Abbreviations: CPR, cardiopulmonary resuscitation; DNR, do not resuscitate.

^a^ Two of us (I.M.O. and E.M.L.) inductively created categories from reviewed excerpts to describe common response patterns. Category definitions were discussed with all authors and further refined. Differences were resolved through iterative discussion until all authors agreed on definitions for each category. Two of us (S.T.V. and M.M.H.) then reread and categorized all excerpts with some excerpts fitting multiple categories.

^b^ Thirty-two intensivists indicated they offered at least 1 choice and did not offer comfort care at this time.

Less common choices included continuing or adding forms of life support (16%) such as dialysis: “…would he want dialysis, do you think?” Three intensivists ( 9%) marked unclear or ambiguous language as highlighting choice. For example, this excerpt, which is a question to the surrogate, was marked as highlighting the existence of a choice: “Has he ever talked to you about what to do if say, his heart were to stop and he was to need somebody to push on his chest to bring his heart back, has he ever talked to you about what he would want in such a situation?”

One intensivist (3%) offered the choice of a palliative care consultation. No discernable choice was offered in the text marked by 14 intensivists (44%). However, most of these statements alluded that the daughter might be offered choices in the future if her father’s clinical condition deteriorated further.

### Qualitative Analysis of Providing a Recommendation

The 33 intensivists (43%) who responded yes to the question, “During the simulation, did you provide a recommendation?” identified a wide range of recommendations in their transcripts (Table 3). The most common recommendation was to continue the current treatment plan (33%). Intensivists either stated they would continue the current plan or recommended a treatment plan to maximize the patient’s chances of survival: “I'd like to try to lighten the sedation and other things that I think we can do that are basically noninvasive and not really big deals. I'd like to do an ultrasound of the heart to try to get an idea about what his fluid status is and we can make a decision about giving fluid, talking with the kidney docs about doing dialysis and trying to take it away.”

**Table 3.** Categorized Recommendations Identified in Text Marked by 33 Intensivists Reviewing Their Own Transcripts Who Responded Yes to the Question, “During the Simulation, Did You Provide a Recommendation?”

Response category^a^	No. (%)^b^	Intensivist response example
Continue current treatment plan	11 (33)	“And so I would recommend that we continue the treatments that we're using right now…”
Withhold CPR	9 (27)	“I strongly recommend to families that we avoid CPR…”
Meet in the future to make decisions	8 (24)	“Well, what's say we also make a plan to meet tomorrow maybe? And that way, after you chat with some of his friends and then family... you know... try to get a better sense of what he would've wanted.”
A specific therapy or procedure	4 (12)	“We may need to put a catheter into either his neck or his groin for dialysis if the nephrology consultants feel that dialysis is indicated at this point.”
Provide or consult with palliative care	2 (6)	“I also want to add the different doctors to the team... a palliative care doctor, if that's okay with you. It's basically a doctor who specializes in palliation in case things don't go well, in case we have to talk about end of life issues.”
Self-care for the surrogate	1 (3)	“So, one of the things I like to tell my patients is you can't help take care of him unless you're also taking care of yourself.”
Comfort-focused care	1 (3)	“I think if one of those things happened that it would... be aligned with what you've told me about him, for us to focus on comfort and make sure that he's suffering as little as possible, rather than continue to do more things to keep him alive while we.”
Unclear	12 (36)	“He may not be able to know that they're here because we're keeping him comfortable with the sedation medicine and that's, of course, a very important goal that we have, so we don't want him to experience any discomfort or pain while we're treating this severe illness, but I think it would be important for the family.”

Abbreviation: CPR, cardiopulmonary resuscitation.

^a^ Two of us (I.M.O. and E.M.L.) inductively created categories from reviewed excerpts to describe common response patterns. Category definitions were discussed with all authors and further refined. Differences were resolved through iterative discussion until all authors agreed on definitions for each category. Two of us (S.T.V. and M.M.H.) then reread and categorized all excerpts with some excerpts fitting multiple categories.

^b^ Thirty-three-intensivists indicated they provided a recommendation. Some responses fit multiple categories.

Nine intensivists (27%) recommended withholding CPR: “but, if he's as sick as this and his heart stops, it just wouldn't make sense to do CPR.” Eight intensivists (24%) recommended meeting again in the near future to reassess or make further plans: “Again, we have 24 hours. You and I will [then] sit down again and discuss.” Less common recommendations included specific therapies such as a thoracentesis or dialysis (12%) and a recommendation for palliative care consultation (6%). One intensivist (3%) recommended comfort care and 1 (3%) recommended self-care for the surrogate. In 12 transcripts (36%) reviewed, the recommendation was unclear. Marked text from these manuscripts often used indirect language about end of life care, such as: “Sort of in the midst of thinking about those bigger picture things is whether maybe we should say well, if something sudden happens like that, we just let him pass comfortably, which is really hard to think about.”

### Blinded Colleague Binary Assessment of Conveying Risk of Death (Prognosis)

After reviewing their own transcripts, 61 intensivists (80%) reported conveying the patient’s risk of death. Blinded colleagues reviewing the same transcripts agreed that 42 (69%) of those 61 had conveyed the patient’s risk of death. Examples of language from transcripts in which there was agreement include, “My concern is that this infection is going to take his life,” and “[there’s a] high chance that he’s not going to make it out of the hospital.” Nineteen intensivists (25%) reported conveying the patient's risk of death but blinded colleagues disagreed, for example: "and because of that and because of the fact that he’s on two different forms of life support, I’m worried about how he’s going to do during this hospitalization. So he’s very, very sick. He’s one of the sicker patients that we have in our ICU.” Two intensivists (3%) who reported that they had not conveyed prognosis for risk of death were rated as having done so by their blinded colleagues. One example of that is, “but, I think it would be important not to ignore that possibility. I'm not sure if he's going to make it through... pull through this one. I'm very concerned about him not being able to pull through this one.” In addition, there was agreement between intensivists and the blinded colleagues that risk of death was not conveyed in 13 transcripts (17%). Blinded colleagues reviewing transcripts were significantly less likely to code that prognosis was conveyed compared with intensivists reviewing their own transcript (odds ratio, 0.10; 95% CI,0.01-0.44; *P* < .001).

### Blinded Colleague Ordinal Assessment of Offering Care Focused on Comfort

Twenty-five intensivists (33%) reported offering comfort care during the simulated family meeting after reviewing their own transcript (Table 4). Blinded colleagues reading the same transcripts rated 1 transcript (4%) as containing a clear offer of care focused on comfort. Colleagues rated offers in 4 transcripts (16%) as understandable, 3 (12%) as vague, and 2 (8%) as offering care focused on comfort in a way they did not believe would be understandable to a family surrogate. The remaining 15 transcripts (60%) were rated as not including an offer of care focused on comfort although some of these transcripts included the words comfort or comfortable. For example, 1 intensivist stated: “And if [he gets worse], the only thing I can promise you… we can keep him comfortable, but I cannot promise we can save his life.”

**Table 4.** Clarity of Comfort Care Offer as Rated by Blinded Intensivists

Blinded intensivist rating of clarity	No. (%) of transcripts (n=25)^a^	Example quotation^b^
Clear	1 (4)	“… we can do therapies that are focused on comfort and work on the shortness of breath or any pain and support him through the illness without trying to keep somebody alive. A lot of people… people often do die in that setting. Some people make that choice…”
Understandable	4 (16)	“Rather than aggressively make sure we treat everything that comes up to aggressively make sure he is comfortable in the time he has left with you and your husband and your children... have as much quality to it as possible.”
Vague	3 (12)	“If it looks very certain that he's going to die, some people say if I got to that point… you know… just make me comfortable. Don't keep doing this if it's not going to lead to anything.”
Not understandable	2 (8)	“My goal would shift from trying to extend my life as long as possible to being pain free for my remaining time.”
Not offered	15 (60)	“Has he ever talked to you about if he thinks he seems to get worse whether he would want to focus more on comfort or aggressive interventions?”

^a^ Twenty-five intensivists (33%) reported that they offered care focused on comfort after reviewing the transcript of their family meeting.

^b^ These quotations were marked by the intensivists as an offer of comfort care by the intensivists reviewing the transcript of their own simulated family meeting.

## Discussion

Attending intensivists in this study reviewed the transcript of a simulated ICU family meeting in which they participated at least 13 months earlier. The simulation scenario involved a patient at high risk of death on ICU day 3. Intensivists were instructed to identify key communication skills for shared decision-making about the care of critically ill patients endorsed by national professional societies. Fewer than half of the intensivists in this study indicated offering a choice or providing a recommendation, and both the nature and framing of choices and recommendations varied widely. Intensivists were also significantly more likely to self-report they conveyed risk of death when compared with blinded colleagues reviewing the same transcripts. In addition, one-third of intensivists reviewing their own transcript reported that they offered care focused exclusively on comfort, but blinded colleagues reading these transcripts disagreed with this assessment more than half the time.

Measuring shared decision-making is difficult and evaluating decision-making at the end of life is particularly challenging. Landmark studies assessing shared decision-making in the ICU^10^ as well as how ICU clinicians discuss patient preferences and values^6,7^ have used trained study personnel to deductively code transcripts of recorded conversations for the presence of specific topics and skills. Instruments and methods for evaluating clinician-patient interactions such as the Observer OPTION 5 scale^12^ and the Roter Interaction Analysis System,^11^ are similar in that trained experts with high interrater agreement review audio- or video-recorded encounters and decide whether predefined acts occurred. Qualitative analysis using inductive coding by experts has also been used to develop theories about how intensivists respond to questions about prognosis,^20,21^ statements about spirituality,^22^ and empathize^23^ during family meetings.

The discrepancies between the communication skills self-identified by intensivists and identified by blinded colleagues in reviewing the same transcripts are notable. It is practical to treat coding by study team members as a reference standard, particularly when they are experts in the field or exhibit high interrater agreement. However, this approach may lack content validity for the physicians enrolled in the study. Moreover, if the goal of evaluating transcripts of patient-clinician interactions is to quantify what was successfully communicated or understood by patients and their proxies, whether research staff and clinicians are capable of assessing this outcome accurately given their familiarity with the topic area and comparative high health literacy is unclear. This question means that expert coding of a transcribed conversation may not reflect what intensivists believe they communicated, or what a patient or their family actually understood.

Our results suggest that methods that ask doctors to self-evaluate shared decision-making face the same potential compromises to validity as patient-reported measures of shared decision-making, namely that physicians may not recognize the encounter as a decision point or agree that a decision exists and that encounters between clinicians and families of ICU patients often include more than one complex decision.^24^ Moreover, methods of evaluating physician communication skills that rely on self-report are vulnerable to the Dunning-Kruger effect, in which people who lack a skill also lack the ability to evaluate competence—including their own competence—in that skill.^25,26,27^ This effect may also prevent a clinician with underdeveloped communication skills from recognizing when they are not adherent with shared decision-making recommendations.

Almost half of intensivists in this study reported that they highlighted a choice during the simulation but did not offer the option of care focused on comfort. In about one-third of these transcripts, the intensivists explained that the surrogate might have a choice in the future if her father’s condition deteriorated further. For example: “If he's not getting better or certainly if he's getting worse over the next few days or a week or so, we're going to certainly have to sit down again and decide how to deal with things.” Similarly, one-third of intensivists who reported that they made a recommendation marked text in which they recommended continuing with current treatment, with another 24% recommending that the conversation be reinitiated in the future. This finding suggests that while critical care guidelines endorse providing the option of care focused on comfort,^17^ intensivists may view these guidelines as applying only after patients have failed lengthy trials of intensive care.^28^

### Limitations

This study has limitations. First, 34% of invited intensivists chose not to participate in this follow-up survey. Second, disagreements between self-assessment and blinded colleague assessment could be attributable to the colleagues harboring unusual views and that interrater reliability was not assessed. Third, nonverbal communication is not reflected in a transcript. Fourth, intensivists may behave differently in simulations than in clinical settings. In addition, we asked intensivists rather than patient surrogates to assess what was communicated to a simulated surrogate with low health literacy. Patient surrogates are better qualified to report this outcome and would ideally be used as a reference standard in this scenario. However, using surrogates with varying degrees of health literacy to interpret lengthy transcripts of simulated encounters is expensive and logistically complicated.

## Conclusions

A year after participating in simulated shared decision-making, intensivists who read a transcript of their simulation were significantly more likely than colleagues reading the same transcript to report they had performed key communication skills endorsed by their professional societies. This finding suggests that evaluations of transcribed clinical interactions for research purposes may not reflect how intensivists experienced or remembered them. More importantly, clinicians who lack communication skills endorsed in professional guidelines may also lack the metacognitive skills required to recognize their deficiencies. In essence, self-assessment may not be possible. This finding makes routine feedback and continuing education on communication and shared decision-making essential at all levels of practice. Without feedback and continuing education, those who would benefit most from supplemental coaching may believe they are already performing recommended skills.
